# Gesture Reduces Mapping Difficulties in the Development of Spatial Language Depending on the Complexity of Spatial Relations

**DOI:** 10.1111/cogs.70046

**Published:** 2025-02-24

**Authors:** Ercenur Ünal, Kevser Kırbaşoğlu, Dilay Z. Karadöller, Beyza Sümer, Aslı Özyürek

**Affiliations:** ^1^ Multimodal Language Department Max Planck Institute for Psycholinguistics; ^2^ Department of Psychology Ozyegin University; ^3^ Department of Psychology Middle East Technical University; ^4^ Department of Linguistics University of Amsterdam; ^5^ Donders Institute for Brain, Cognition and Behaviour Radboud University

**Keywords:** Spatial language, Gesture, Multimodal language, Language development, Spatial cognition

## Abstract

In spoken languages, children acquire locative terms in a cross‐linguistically stable order. Terms similar in meaning to *in* and *on* emerge earlier than those similar to *front* and *behind*, followed by *left* and *right*. This order has been attributed to the complexity of the relations expressed by different locative terms. An additional possibility is that children may be delayed in expressing certain spatial meanings partly due to difficulties in discovering the mappings between locative terms in speech and spatial relation they express. We investigate cognitive and mapping difficulties in the domain of spatial language by comparing how children map spatial meanings onto speech versus visually motivated forms in co‐speech gesture across different spatial relations. Twenty‐four 8‐year‐old and 23 adult native Turkish‐speakers described four‐picture displays where the target picture depicted in‐on, front‐behind, or left‐right relations between objects. As the complexity of spatial relations increased, children were more likely to rely on gestures as opposed to speech to informatively express the spatial relation. Adults overwhelmingly relied on speech to informatively express the spatial relation, and this did not change across the complexity of spatial relations. Nevertheless, even when spatial expressions in both speech and co‐speech gesture were considered, children lagged behind adults when expressing the most complex left‐right relations. These findings suggest that cognitive development and mapping difficulties introduced by the modality of expressions interact in shaping the development of spatial language.

## 1 Introduction

Children become increasingly adept at describing what is around them, including objects and their locations. Describing object locations requires identifying the object to be located, the object that serves as the reference, and creating a mental representation of the spatial relation between the two. Then, children need to map this representation onto the spatial terms available in their language. This is a relatively early emerging skill that nevertheless follows a lengthy developmental timetable.

There is considerable debate about the precise factors involved in this timetable. One explanation is that the way children use (or not use) certain terms in language reflects their cognitive understanding of the concepts expressed by them (Clark, 1973b; Dromi, 1987; Smiley & Huttenlocher, 1995). For instance, earlier emergence of the spatial terms similar in meaning to *in* and *on* has been assumed to reflect earlier understanding of the notions of containment and support (Johnston & Slobin, 1979; Piaget & Inhelder, 1967). On the other hand, later emergence of the terms similar in meaning to *front*, *behind*, *left*, and *right* have been attributed to the complexity of the computations involved in locating objects with respect to each other and in accordance with a viewpoint (Piaget, 1972). According to another view, the way children use terms in language is not only determined by their cognitive capacity to entertain the concepts expressed by them but also by the features of the linguistic terms that may make recognizing the mappings between certain meanings and the terms expressing them easier or harder (Gleitman, 1990; Gleitman, Cassidy, Nappa, Papafragou, & Trueswell, 2005; Papafragou, Cassidy, & Gleitman, 2007). This mapping challenge is also recognized by Landau and Jackendoff who proposed that “to account for language about space, there must be a translation between spatial representations and language” (1993, p. 218). One possibility is that the spatial terms in speech that have arbitrary links to the spatial meaning that they express might make this translation particularly difficult. This is plausible according to research on co‐speech gestures that exploit different representational resources than speech. This work shows that co‐speech gestures ease the expression of several concepts that have rich visual and spatial features (Alibali & Goldin‐Meadow, 1993; Alibali, Kita, & Young, 2000; Church & Goldin‐Meadow, 1986). Nevertheless, this evidence comes from domains that are cognitively challenging in general. Thus, how variations in complexity within a single domain influence mapping meaning onto expressions across different modalities is overlooked. Here, we revisit the discussion on the contribution of cognitive and mapping difficulties in the domain of spatial language. We pick this domain as it provides an excellent test case for examining how children map spatial meanings onto arbitrary forms in speech as opposed to visually motivated forms in gesture while systematically comparing spatial relations that vary in their complexity.

In the sections that follow, we report an experiment investigating how Turkish‐speaking children and adults express different locative spatial relations (i.e., in‐on, front‐behind, left‐right) in multimodal descriptions in speech and co‐speech gesture. Before introducing the specific hypotheses motivating our investigation, we review prior work on the acquisition of spatial language and its relation to complexity of the relations as well as the role of gestures in the expression of visual‐spatial information.

### 1.1 The acquisition of locative terms

It is widely acknowledged that in spoken languages children acquire locative terms in a stable order across languages (e.g., Clark, 1980; Johnston, 1984; Johnston & Slobin, 1979; see Grigoroglou & Papafragou, 2019b for an overview). A cross‐linguistic study with young learners of English, Italian, Serbo‐Croatian, and Turkish shows that children start using locative terms similar to *in*, *on*, and *under* the earliest, around age 2 (Johnston & Slobin, 1979). This is followed by terms similar to *front* and *behind* (English and Greek: Grigoroglou, Johanson, & Papafragou, 2019; English: Johnston, 1984; English, Italian, Serbo‐Croatian, and Turkish: Johnston & Slobin, 1979; see also Kuczaj & Maratsos, 1975; Levine & Carey, 1982). Children's earliest uses of these terms are in reference to their own body. Later, they begin using *front* and *behind* to refer to objects that have intrinsic front and behind sides (e.g., a teddy bear, a car) and then extend them to refer to objects that do not have intrinsic front and behind sides (e.g., a box). Locative terms similar to *left and right* emerge the latest and may not be fully acquired until age 10 (Tseltal and Spanish: Abarbanell & Li, 2021; English: Harris, 1972; Rigal, 1994, 1996; Turkish: Karadöller, Sümer, Ünal, & Özyürek, 2024; Sümer, 2015; see also Benton, 1959; Piaget, 1972). Children typically use locative terms similar to *side* or *next to* in order to express front‐behind or left‐right relations before they fully acquire the terms similar to *front‐behind* or *left‐right* (English: Abarbanell & Li, 2021; Johnston, 1984; Turkish: Karadöller et al., 2024; Sümer, Perniss, Zwitserlood, & Özyürek, 2014). However, since these terms fail to convey the exact spatial relation between the objects, they cannot distinguish one relation from the other (e.g., left vs. right) and can be underinformative.

### 1.2 Cognitive difficulties in the development of spatial language

What might account for the developmental patterns in the acquisition of spatial language? One possibility is that the order of acquisition of locative terms in spoken languages is explained by the complexity of the spatial relations expressed by these terms. In this view, the acquisition of locative expressions is made possible by conceptual development (Johnston & Slobin, 1979) which determines “both their order of emergence and changes in their meanings” (Johnston, 1984, p. 421). This is consistent with a broader view on language acquisition according to which language learning involves mapping linguistic input onto pre‐existing conceptual representations (Arunachalam & Waxman, 2010; Hespos & Spelke, 2004), such that patterns of lexical emergence can be taken as a strong indicator of the underlying conceptual representations (Clark, 1973b; see also Dromi, 1987; Huttenlocher, Smiley, & Charney, 1983; Smiley & Huttenlocher, 1995).

According to this view, terms similar to *in* and *on* are acquired earlier as containment and support relations expressed by these terms is less complex than the relations expressed by terms similar to *front*, *behind*, *left*, and *right*. This is especially the case for relations that hold between objects that do not themselves provide an intrinsic front‐behind or left‐right axis. In these cases, the axes and specific sides are defined by projecting the viewer's perspective onto the larger and relatively more stable ground object that serves as the reference (Levinson & Wilkins, 2006; Shusterman & Li, 2016). Then, the smaller and relatively more mobile figure object is located according to this projection on the ground object. These extensions or projective uses of front, behind, left, and right relations all depend on a viewpoint. Different types of viewpoint‐dependent relations also vary in terms of their complexity. Sagittal viewpoint‐dependent relations include features such as visibility (for front) and occlusion (for behind) that distinguish one relation from the other and can be considered relatively less complex. However, lateral viewpoint‐dependent relations expressed by terms similar to *left* and *right* are symmetrical and harder to distinguish from one another. Therefore, lateral relations can be considered relatively more complex (Landau & Jackendoff, 1993; Shusterman & Li, 2016).

This view on the acquisition of locative terms is supported by several pieces of empirical evidence. First, the order of acquisition of locative terms in speech is cross‐linguistically consistent despite the variations in the characteristics of the formal devices in spoken languages used to express location information (e.g., morphology, lexical diversity, syntax) (Johnston & Slobin, 1979). Second, studies directly comparing children's acquisition of locative terms in language and nonlinguistic understanding of the relations expressed by them show that children show some understanding of spatial relations before they express the same relations in language (Clark, 1973a; Levine & Carey, 1982). Finally, spatial relations that emerge earlier in speech are also discriminated earlier by prelinguistic infants compared to the relations that emerge later in language (e.g., containment; Casasola, Cohen, & Chiarello, 2003; Hespos & Baillargeon, 2001b; and support; Baillargeon, Needham, & Devos, 1992; Casasola & Cohen, 2002; e.g., occlusion Hespos & Baillargeon, 2001a; see Casasola, 2008; and Quinn, 2007 for an overview).

### 1.3 Mapping difficulties in the development of spatial language

An additional possibility is that the acquisition of locative terms in spoken languages presents mapping problems for children. That is, children may be delayed in expressing certain spatial meanings not only because of the complexity of the cognitive computations involved in extracting the spatial relations but also because of the difficulty in discovering the correspondence between locative terms in spoken language and spatial relation they express. Several factors may contribute to the difficulty of these form‐to‐meaning mappings.

Previous work with spoken languages typically focuses on the role of linguistic factors (e.g., Bowerman, 1996; Bowerman & Choi, 2001; Levinson & Wilkins, 2006). These factors include the formal devices in language used for expressing location (e.g., adpositions, case‐marking, verbs) and the way languages carve up the semantic space of location. For instance, English‐speaking children who have to learn a single preposition (*on*) to encode support begin expressing support relations earlier than Dutch‐speaking children who have to learn a more complex three‐way system that encodes different types of support (*op*: support from below, *aan*: hanging support, *om*: encirclement) (Gentner & Bowerman, 2009).

Another factor that might contribute to the difficulty of these form‐to‐meaning mappings—especially in the domain of space—is the requirement to transform modality‐specific visual‐spatial information into discrete and categorical forms in speech that have arbitrary links to the spatial meaning they express. In fact, recent evidence on the development of spatial expressions in sign languages that exclusively rely on the visual modality has shown that signing children informatively express left‐right relations in sign more frequently than age‐matched speaking peers do so in speech (Karadöller et al., 2024; Sümer, 2015). Therefore, part of the difficulty of the mapping problem might come from the affordances of the modality used for expressing spatial information.

Gestures also have different affordances than speech for representing and packaging visual‐spatial information (Kita, Alibali, & Chu, 2017). That is, they have visually motivated links to the spatial meaning that they express. For instance, speakers may indicate that an object is to the left of another object by pointing to the left side of their gesture space or by placing their hands in different sides of their gesture space to indicate the relative locations of objects with respect to each other. In such cases, how gestures are used to represent object locations maps onto how objects are located in real space in an analog way. These affordances might ease the expression of spatial information in gesture (cf. Goldin‐Meadow, 1999).

The idea that visually motivated nature of gestures may ease the difficulty of the mapping problem is plausible based on previous evidence. Children frequently use gestures to express information not found in accompanying speech. These nonredundant gestures are especially seen in domains that are rich in visual‐spatial information, such as geometry (Calero, Shalom, Spelke, & Sigman, 2019), spatial directions (Austin & Sweller, 2014; Sauter, Uttal, Alman, Goldin‐Meadow, & Levine, 2012; Sekine, 2009), caused motion events (Furman, Küntay, & Özyürek, 2014), and instruments of causal actions (Göksun, Hirsh‐Pasek, & Golinkoff, 2010). In a particularly relevant demonstration, Karadöller and colleagues (2024) showed that this is also the case for describing object locations involving left‐right relations. Recall that children younger than 10 are typically underinformative when describing left‐right relations. In that study, 8‐year‐old Turkish speakers often referred to left‐right relations using general locative terms that correspond to *side* or *next to*. However, the same children frequently supplemented such spoken descriptions with gestures that conveyed the relative locations of the objects. Hence, children's co‐speech gestures disambiguated underinformative speech, and their multimodal spatial expressions were informative.

### 1.4 Open questions

The work just reviewed suggests that visually motivated spatial expressions in gesture can ease the mapping problem—at least to some extent—such that children can convey spatial information missing from accompanying speech via gestures. However, this work leaves open several key issues that need to be addressed to more precisely estimate the contributions of cognitive and mapping difficulties in the development of spatial language. On the one hand, studies have investigated the order of emergence of locative terms in spoken language and made claims about cognitive difficulties without considering modality of expression as a potential source of difficulty (e.g., Clark, 1973a; Johnston & Slobin, 1979). On the other hand, evidence for the facilitative role of gesture for the expression of visual‐spatial information comes from domains that are cognitively complex in general but does not account for the variations driven by complexity within a single domain (e.g., Austin & Sweller, 2014; Calero et al., 2019; Göksun et al., 2010; Karadöller et al., 2024). It is important to consider the contributions of cognitive and mapping difficulties simultaneously from the perspective of both cognitive and language development.

From the perspective of cognitive development, children's gestures have been taken as a window into their underlying thinking in many cognitive tasks (mathematical equivalence: Alibali & Goldin‐Meadow, 1993; Broaders, Cook, Mitchell, & Goldin‐Meadow, 2007; Piagetian conservation: Alibali et al., 2000; Church & Goldin‐Meadow, 1986). In such tasks, gestures supplement speech and hence uncover the knowledge that they implicitly have but cannot yet express in speech. Importantly, such uses of gesture typically precede the upcoming changes in cognitive development, indicating that children might be in a stage of transitional knowledge (Alibali & Goldin‐Meadow, 1993; Church & Goldin‐Meadow, 1986; Goldin‐Meadow & Alibali, 2013). One open question is how this role of gesture changes throughout cognitive development during versus after the transitional knowledge stage.

For language development, it is important to consider that although gesture relies on a different modality of expression than speech, speech and gesture together form an integrated multimodal system (Holler & Levinson, 2019; Özyürek, 2014; Trujillo & Holler, 2023; for an alternative view, see Tomasello, 2010). Thus, whether and how children express certain concepts in speech may influence the use and function of the accompanying gestures. In fact, longitudinal evidence on early language development shows that children use (pointing) gestures to refer to certain objects before they do so in speech, but once they learn to refer to them in speech, they are less likely to gesture about the same referents (Iverson & Goldin‐Meadow, 2005). Even though the opposite pattern (i.e., referring to objects initially in speech and later transferring to gesture) is possible (e.g., iconic gestures about actions follow verbs in speech Özçalışkan, Gentner, & Goldin‐Meadow, 2014), it is much less likely (Karadöller et al., 2024). Therefore, children's early uses of gestures seem to be sensitive to their expressions in speech. How this changes later in language development and across concepts that have already been versus not yet mapped onto speech remains to be seen. For instance, gestures might be frequently used for supplementing speech for the most complex left‐right relations that are not yet expressed in speech, but not necessarily or to a lesser extent for less complex relations (e.g., front‐behind or in‐on) that are (more) frequently expressed in speech.

### 1.5 The present study

Our goal in the present study is to contribute to the discussion on how cognitive and mapping difficulties are implicated in children's ability to describe object locations in informative ways. To do so, we investigate how Turkish‐speaking children and adults describe object locations by expressing different spatial relations in speech and gesture. To elicit spatial descriptions, we used a communication task. Participants described a target picture among a set of four pictures to an addressee. Each of the four pictures depicted the same figure (the object to be located) and ground (reference) objects, but in a different spatial relation. Thus, participants had to express the exact spatial relation between the objects to communicate the target picture to the addressee in an informative way. For the spatial relation in the target picture, we used three different locative spatial relations that vary in their complexity: topological relations in‐on (the least complex), sagittal relations front‐behind, and lateral relations left‐right (the most complex). For sagittal and lateral relations, we focused on relations that hold between objects that do not have intrinsic front, behind, left, or right sides as these emerge the latest in acquisition (Johnston & Slobin, 1979; Levine & Carey, 1982).

We focused on Turkish as it provides a particularly straightforward case for addressing our goal. Turkish encodes locative relations using post‐positional phrases. Post‐positions are derived by adding a possessive suffix and a locative case marker to spatial nouns (e.g., *iç‐in‐de* = at the inside of; see coding section for more information) (Göksel & Kerslake, 2011). Specific (and single) spatial nouns express the notions of containment (*iç*) and support (*üst*), visibility (*ön*) and occlusion (*arka*) in the sagittal axis, and symmetrical relations in the lateral axis (*sol‐sağ*). Additional spatial nouns can express proximity (*yakın* similar to *next to*) or axial parts (*yan*, *taraf* similar to *side*). Note that in Turkish, there are a wider range of alternatives that can be considered as underinformative for viewpoint‐dependent relations. Importantly, post‐positional phrases with locative terms corresponding to *in*, *on*, *front*, *behind*, *left*, and *right*, as well as the underinformative terms *side* and *next* to, are derived in the same way and hence similar in terms of their morphological complexity. Furthermore, in Turkish, the underinformative locative terms corresponding to *side* or *next to* can be used for describing both sagittal and lateral viewpoint‐dependent relations (Sümer et al., 2014), which differ in their complexity. Thus, our experimental setup and choice of language allows us to manipulate the complexity of the spatial relation while controlling for additional factors, such as pragmatic demands of the task, morphological complexity of location terms, and the relations for which the underinformative term is likely used. Therefore, changes in the use of gestures to convey spatial information missing from speech can be attributed to the complexity of the relations and not to any other factors.

As a first step, we aimed to replicate previously reported patterns for the order of acquisition of locative terms in speech. For children, we expected the frequency of informative expressions in speech to change across the complexity of spatial relations. Specifically, children should produce informative expressions in speech most frequently—and in similar frequencies as adults—for in‐on, followed by front‐behind, and least frequently for left‐right. Adults were expected to produce informative expressions in speech overwhelmingly regardless of the complexity of the spatial relations.

Assuming that the previously reported patterns for the order of acquisition of locative terms would surface in the speech of children, we further investigated spatial expressions in gestures together with those in speech. We tested whether the main modality used for informatively expressing the spatial relation changes across the complexity of the spatial relations. If so, children should be more likely to rely on gesture as opposed to speech to informatively express more complex spatial relations; but this pattern should reverse for less complex spatial relations that have already been mapped onto speech. Adults, on the other hand, are expected to rely on speech regardless of the complexity of spatial relations and, therefore, produce many fewer expressions that become informative with gestures.

Finally, we asked whether there would be an interaction between the complexity of spatial relations and age. That is, we tested if developmental differences between adults and children in producing informative descriptions of object locations found for speech would diminish when spatial expressions in gesture were also considered and whether this would be modulated by the complexity of the spatial relations. If so, any developmental differences between children and adults should diminish to a greater extent—and possibly disappear completely—for relatively less complex front‐behind relations. However, we expected developmental differences to diminish but still persist for the most complex left‐right relations. This would indicate that influence of complexity of the relations generalizes to gesture—at least to some extent.

## 2 Method

The methods reported in this study were approved by the Humanities Ethics Assessment Committee of Radboud University and the Ministry of Education in Istanbul, Turkey.

### 2.1 Participants

Data were collected from 47^1^ monolingual native speakers of Turkish in two age groups: children (*n* = 24; 14 females, mean age = 8;6; range = 6;7−9;5) and adults (*n* = 23; 14 females, mean age = 35;9; range = 19;8−50;0). All had learned Turkish from birth and as their first language and were not proficient in another language. For children, we focused on this age group because previous work with Turkish‐speakers showed that this age group still frequently uses the underinformative spatial term corresponding to *side* for both front‐behind and left‐right (Sümer et al., 2014). Children were recruited from third‐year students in one public school in Istanbul, Turkey. Data from seven additional participants (three children) were discarded. Three of these participants (one child) were bilingual. Four of these participants (two children) failed to describe more than 30% of the items. All exclusions were made prior to data analyses. Consent was obtained from adult participants themselves and parents of child participants. Children received a pencil kit as compensation. Adults were given monetary compensation.

### 2.2 Stimuli

**Fig. 1:**
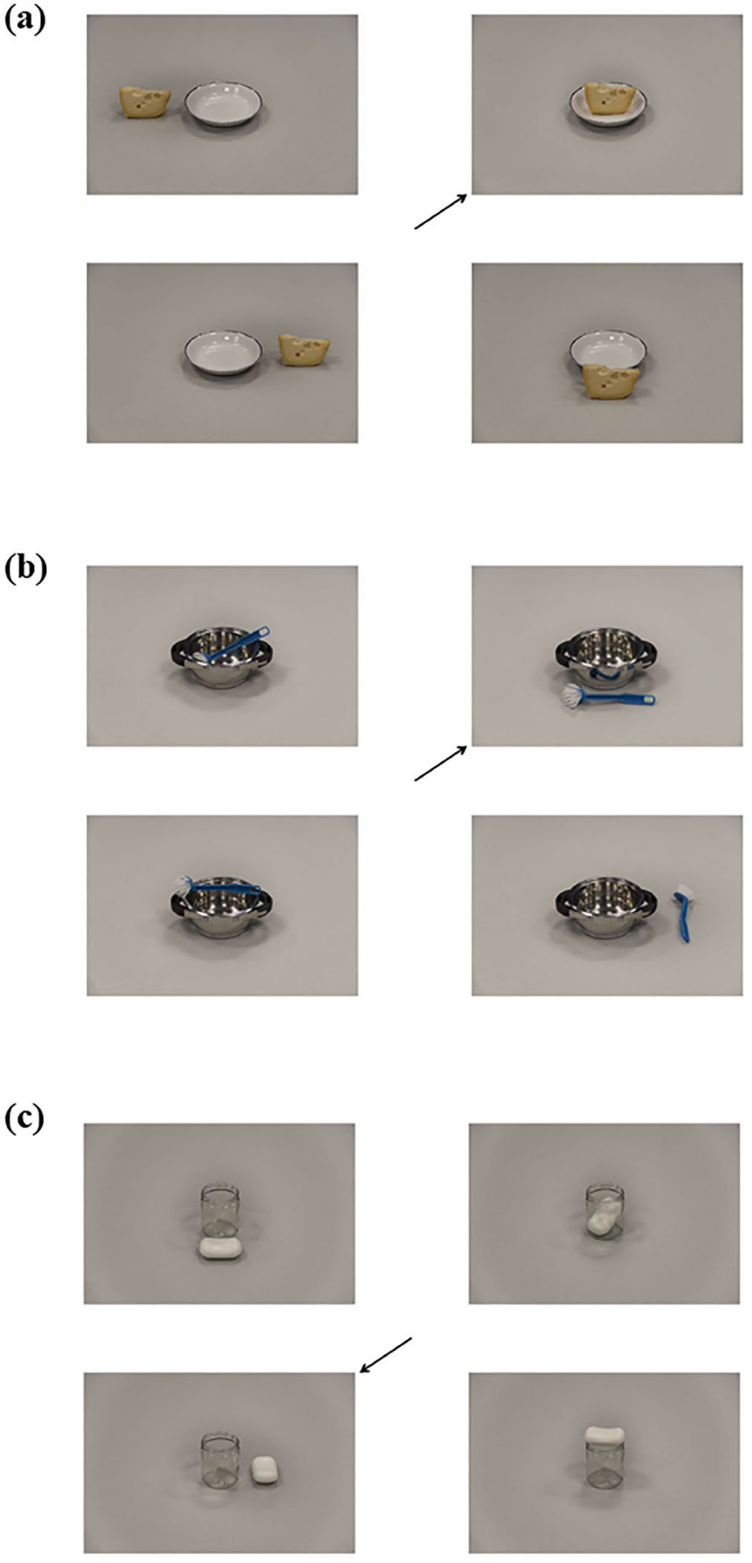
Examples of displays in which the target picture depicts a (a) topological, (b) sagittal, and (c) lateral relation.

The stimuli consisted of two sets of 84 displays depicting four pictures (see Fig. 1). Each picture within a display depicted the same everyday ground and figure objects in a different spatial relation. Ground objects (e.g., jar) did not have any intrinsic sides (e.g., front, behind, etc.) that could be determined by their shape. They were placed in the center of the picture and remained in the same location in all four pictures. Figure objects (e.g., soap) were placed in different locations with respect to ground object in each of the four pictures. Thus, the only distinguishing feature of individual pictures within a display was the spatial relation between figure and the ground objects.

In each display, one picture was designated as the target picture (indicated by the arrow, see Fig. 1) that the participants had to describe to an addressee. Target pictures depicted one of the three types of spatial relations between the objects: topological relations (14 in, 14 on), sagittal viewpoint‐dependent relations (14 front, 14 behind), and lateral viewpoint‐dependent relations (14 left, 14 right). Half of the displays in each set were contrastive: one of the nontarget pictures also depicted a relation from the same type of spatial relation as the target picture. For example, when the target picture depicted a front relation (i.e., sagittal), one of the nontarget pictures depicted a behind relation (i.e., also sagittal), and the other two nontarget pictures depicted topological and lateral relations. The other half of the displays in each set were noncontrastive: nontarget pictures did not depict a relation from the same category as the target picture. For example, when the target picture depicted a front relation, there were no nontarget pictures depicting a behind relation and all three nontarget pictures depicted topological and lateral relations. (See Supplementary Material for more information.)

Across the two sets of displays, each figure object (e.g., the soap) was presented only once. Each ground object (e.g., the jar) was presented four times in total across all displays, but each time together with a different figure object (e.g., jar–soap, jar–razor, jar–whistle, and jar–jar lid). The two sets of displays were equivalent such that the ground objects presented in each display and the spatial relations depicted between the objects were the same. The only difference between the sets were the individual figure objects presented with the ground objects. Half of the participants described the first set of displays, and the other half of the participants described the second set.

Displays were presented in a different pseudorandomized order for each participant with the following constraints: the same ground object did not appear in two or more consecutive trials and the target object did not depict the same type of spatial relation in two or more consecutive trials. Within each display, the assignment of individual pictures to a location (top/bottom left/right) on the screen was also randomized.

### 2.3 Procedure

Each participant was tested in a quiet room in Turkish by a native speaker. Participants were seated across a DELL Precision M4800 laptop. The experiments were run through Presentation® software (Version 16.4, Neurobehavioral Systems, Inc., Berkeley, CA).

Participants saw 84 displays presented on a computer screen. Each trial started with a fixation cross presented in the center of the screen for 2000 ms followed by a display containing four pictures presented for 1000 ms. Next, an arrow pointing to one of the four pictures (i.e., target picture) appeared on the screen and disappeared after 500 ms. The display of four pictures remained on the screen for an additional 2000 ms until a visual white noise screen was presented. Participants were asked to describe the target picture to an adult confederate addressee during the visual white noise screen. This was done to ensure that participants would indeed describe the picture and not merely point to the screen. After the participants finished describing the target picture, they pressed ENTER to initiate the next trial. (See Supplementary Material for exact instructions.)

The addressee's task was to find the target picture among the same display of four pictures on their tablet based on the participants’ description. Participants were informed that the addressee also had the same four pictures on her tablet, but they were arranged in a different way and no arrow was shown. We included an interactive addressee who had to use the information provided by the participant to perform a simple task to maximize the informational needs (Bahtiyar & Küntay, 2008; Grigoroglou & Papafragou, 2019a).

At the beginning of the experiment, there were three practice trials to familiarize the participants with the task. For these trials, another set of three displays that were similar to the ones used in the actual experiment were used. The instructions and the structure of the trials were the same as in the actual experiment. During the practice trials, if participants failed to follow the instructions, the experimenter repeated them. The confederate addressee did not provide any feedback on whether the description was correct to the participants during the practice trials or the experiment. When the participants’ description was missing the spatial relation between the figure and the ground, the addressee asked for the location of the figure (“Where is [Figure object]?”) only once. No other feedback was provided. To ensure that participants would produce gestures spontaneously, they were not given any instructions related to gesture use. Participants were video‐taped with two Canon video cameras from front and side‐top angles for later coding of speech and gestures. This task lasted approximately 20 min.

### 2.4 Coding

Descriptions of target pictures were transcribed and coded for spatial expressions in speech and gesture by native speakers of Turkish using ELAN software (Lausberg & Sloetjes, 2009). For speech, we considered whether and how the spatial relation between the figure and the ground objects were expressed in the entire description. For gesture, we segmented gesture strokes (i.e., the most meaningful part of the hand movement) that accompanied speech and represented location of the figure and/or ground objects (Kita, van der Hulst, & van Gijn, 1998). Nonrepresentational gestures that did not convey any meaning (e.g., beat gestures) or representational gestures that only depicted the shape of the objects without locating them on the gesture space were not included. Gestures always accompanied speech. Speakers could represent the relative location of the figure and/or ground objects using directional pointing gestures, or by placing their hands in the gesture space in relation to each other with either neutral or specific handshapes (49.4% of the gestures consisted of a mixture of these strategies, 33.6% consisted of only pointing gestures, and 16.8% consisted of only placement gestures). Participants represented the location of the objects with gestures from their own viewpoint. In order to ensure reliability, 25% of the gesture data were coded by another native Turkish speaker for the presence of a spatial gesture. There was substantial agreement between the coders in terms of the presence of a spatial gesture (agreement = 88.5%, κ = 0.757, *z* = 21.9, *p* < 001). Based on whether and how spatial information was expressed in speech and gesture, the following categories were created.

**Fig. 2:**
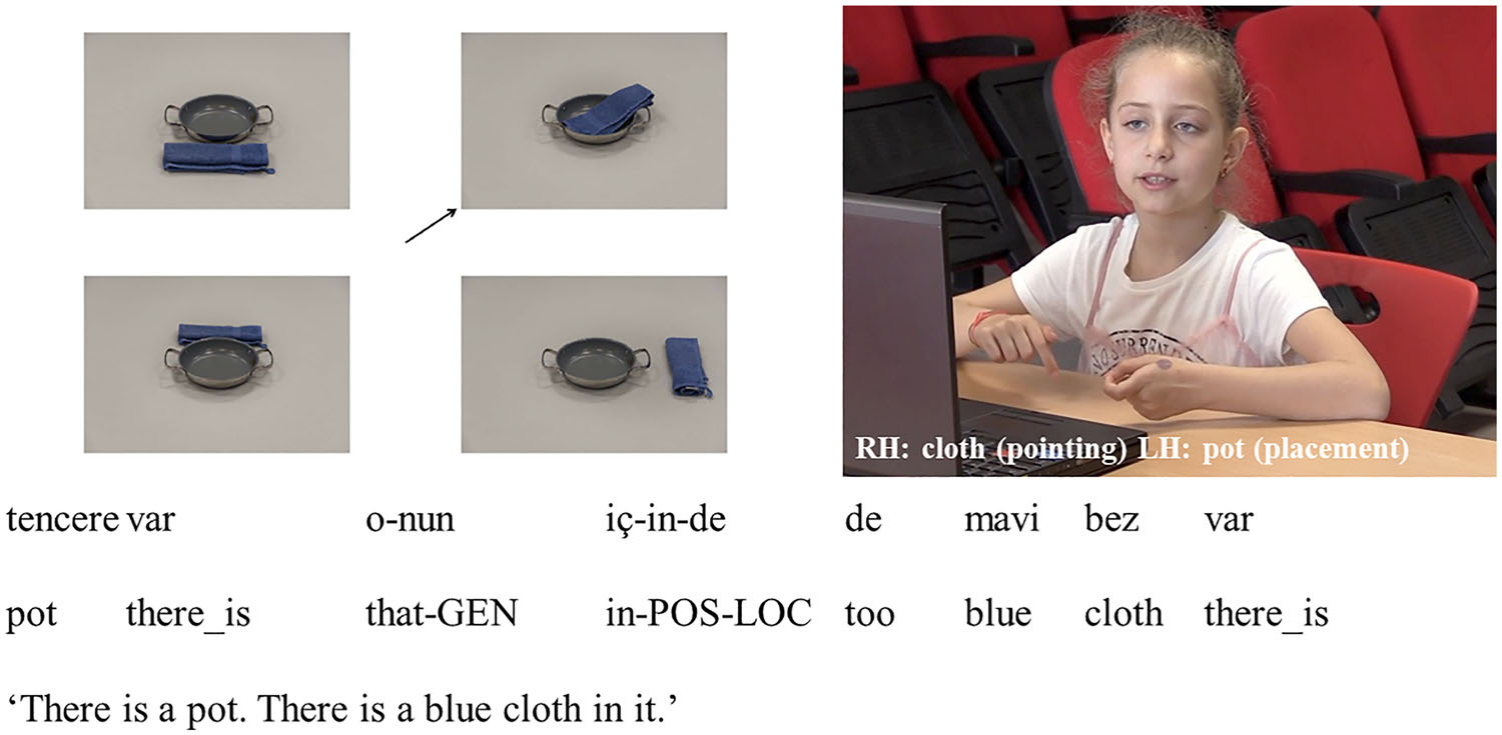
Spatial expression informative in speech.

Descriptions were coded as ***informative in speech*** if participants correctly used specific spatial terms to refer to the target spatial relation in speech. These included: *içinde* (corresponding to inside of), *üstünde* (corresponding to on top of), *önünde* (corresponding to in front of), *arkasında* (corresponding to behind of), *solunda* (corresponding to left of), and *sağında* (corresponding to right of). Some of these descriptions were accompanied by spatial gestures (see Fig. 2 for an example). Even though these gestures could potentially express additional information about visual features of the objects (e.g., size, shape), they were redundant with respect to the expression of spatial information as speech already uniquely distinguished the target spatial relation from the other relations in the display and was informative.

**Fig. 3:**
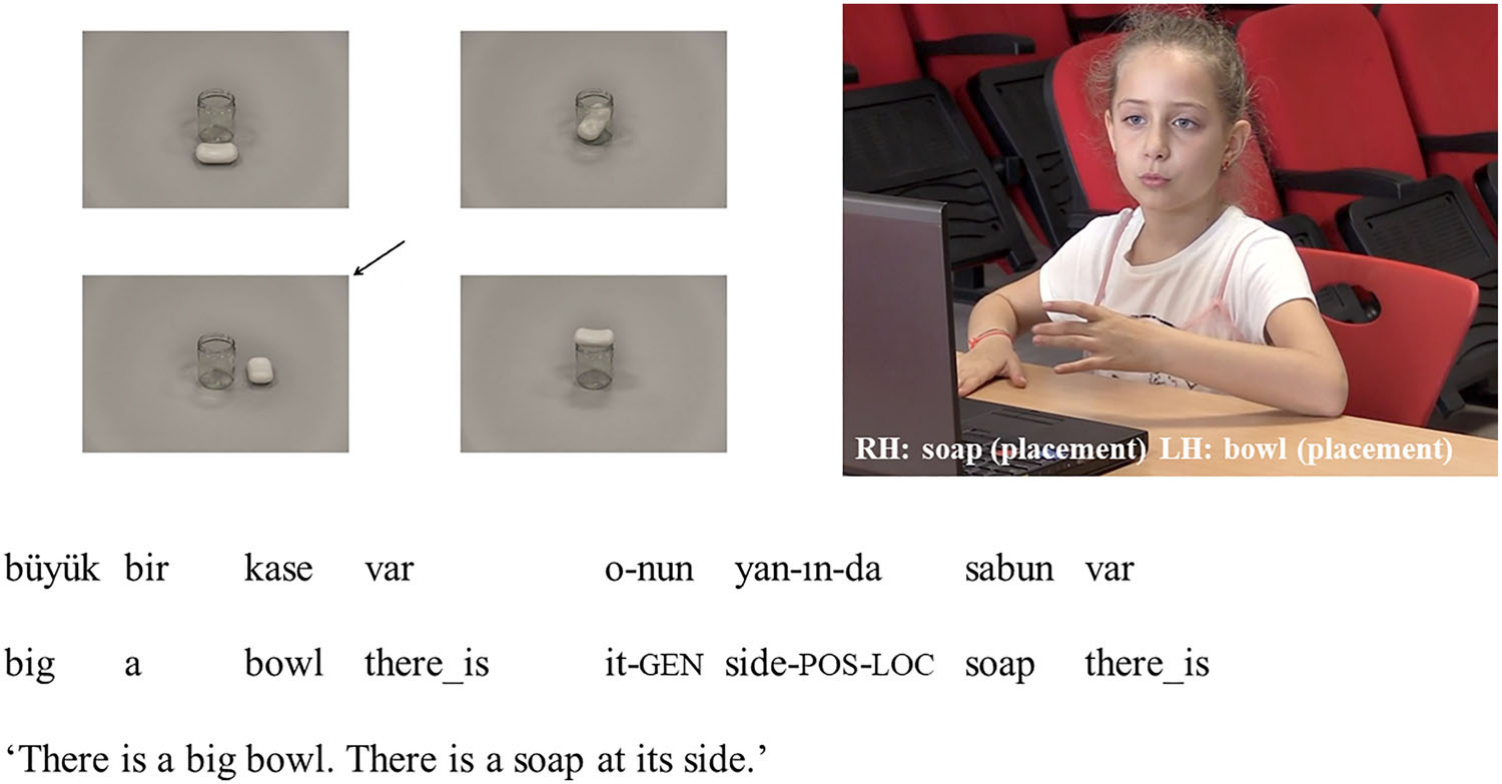
Spatial expression informative with gesture.

Descriptions were coded as ***informative with gesture*** if participants referred to the target spatial relations with general spatial terms in speech, accompanied by pointing or placement gestures that disambiguated the relative locations of the figure and ground objects. The general spatial terms in this category included: *ortasında* (corresponding to at the middle of) for topological relations and *yanında* or *tarafında* (corresponding to at the side of), *yakınında* (corresponding to next to) for sagittal and lateral relations. For these descriptions, speech failed to distinguish the target spatial relation from the other relations in the display and hence was underinformative. For example, using the term *side* to describe the target in Fig. 3 is underinformative, since in Turkish this term can be used to describe both the relation depicted in the target picture (right) and a relation depicted in one of the distracter pictures (front). However, when gesture is considered together with speech, the relative locations of the figure and ground objects are conveyed. Hence, the description becomes informative with gesture.

There were two types of descriptions that fell under the ***underinformative descriptions*** category. These included descriptions that merely labeled the figure and ground objects but not the relation between them and descriptions that referred to the target spatial relation with the general spatial terms in speech (e.g., *side*/*yanında*) without any accompanying gesture. Note that a very small portion of the descriptions that merely labeled the objects were accompanied by gestures (0.4% of the data). However, these descriptions were still underinformative because the relative locations of the figure and ground objects were not conveyed in the multimodal description.

In addition to these three categories, there were also descriptions that did not correctly express the spatial relation between the figure and ground object (e.g., describing the target in Fig. 1c as “*the jar is on the right of the soap*”). These descriptions were 11.7% of the data and were not included in the analysis.

## 3 Results

Speech and gesture production data were analyzed using generalized mixed‐effects logistic regression modeling. Models were fit using *glmer* function of the *lme4* package (version 1.1.17; Bates, Mächler, Bolker, & Walker, 2015) in R (version 4.2.2; R Core Team, 2024). Parameter estimates and significance levels for pairwise comparisons with corrections for multiple comparisons were obtained using *emmeans* (version 1.8.4‐1; Lenth, 2023) and *multcomp* (version 1.4‐22; Hothorn, Bretz, & Westfall, 2008) packages. Figures were produced using *ggplot2* package (version 3.4.1; Wickham, 2016). Data and analysis code are available at https://osf.io/njufw/.

### 3.1 Spatial expressions informative in speech

**Fig. 4:**
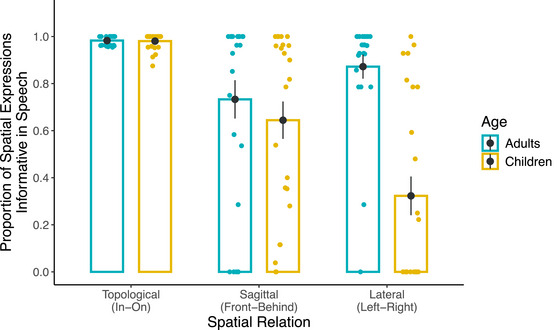
Proportion of spatial expressions informative in speech across spatial relations and age groups. *Note*. Bars and black dots represent group means. Error bars represent standard error of the group means. Colored dots represent participant means.

First, we tested to what extent spatial descriptions conform to previously reported patterns for the order of acquisition such that the frequency of spatial expressions informative in speech would change across the complexity of the spatial relations, especially for children. Fig. 4 shows the proportion of expressions informative in speech out of all spatial expressions for topological, sagittal, and lateral relations across adults and children. A *glmer* model tested the fixed effects of Spatial Relation (topological, sagittal, lateral) and Age (adults, children) on binary values for the presence of expressions informative in speech (1 = present, 0 = absent) at the trial level as the dependent measure.^2^ The fixed effect of Age was tested with centered contrasts (adults coded as −1/2, children coded as +1/2). The fixed effect of Spatial Relation was tested with two planned contrasts. The first contrast compared topological relations that were not viewpoint dependent to sagittal and lateral relations that were both viewpoint dependent (topological coded as −2/3, sagittal coded as +1/3, lateral coded as +1/3). The second contrast compared the two types of viewpoint‐dependent relations (topological coded as 0, sagittal coded as −1/2, lateral coded as +1/2). For the random effects structure, we started maximal (following Barr, Levy, Scheepers, & Tily, 2013) and included random intercepts for Subjects and Items and random slopes for fixed effects that varied within Subjects or Items—in this case, only random slopes for Spatial Relation by Subjects were appropriate (since Age varied between Subjects and Spatial Relation varied between Items). This model produced a singular fit error, indicating that the model was overfitted and that the random effects structure of the model was too complex. Therefore, we gradually simplified the model by first removing random slopes for Spatial Relation by Subjects and then random intercepts for Items until the singular fit error was eliminated. The final model included random intercepts for Subjects only.

The model revealed that the interaction between Age and Spatial Relation was significant for both contrast levels (Topological vs. Sagittal/Lateral: *β* = −2.692, SE = 0.511, *z* = −5.270, *p* < 001; Sagittal vs. Lateral: *β* = −2.713, SE = 0.295, *z* = −9.189, *p* < 001). Follow‐up tests of pairwise comparisons with corrections for multiple comparisons revealed that children produced expressions informative in speech more frequently for topological relations than for both sagittal relations (*β* = 4.004, SE = 0.368, *z* = 10.88, *p* < 001) and lateral relations (*β* = 6.607, SE = 0.395, *z* = 16.72, *p* < 001). Within viewpoint‐dependent relations, they produced expressions informative in speech more frequently for sagittal relations than lateral relations (*β* = 2.603, SE = 0.195, *z* = 13.36, *p* < 001). Interestingly, adults also produced more expressions that were informative in speech for topological relations compared to both sagittal (*β* = 2.669, SE = 0.381, *z* = 6.998, *p* < 001) and lateral relations (*β* = 2.559, SE = 0.361, *z* = 7.098, *p* < 001). However, there were no differences between the two types of viewpoint‐dependent relations for adults (*β* = 0.109, SE = 0.221, *z* = 0.496, *p* = 870). Furthermore, children were adult‐like for topological relations (*β* = 0.067, SE = 0.796, *z* = 0.085, *p* = 932) but not for sagittal (*β* = 1.403, SE = 0.663, *z* = 2.1165 *p* = 034) or lateral relations (*β* = 4.116, SE = 0.660, *z* = 6.238, *p* < 001). These patterns are largely consistent with previously reported developmental patterns from other languages on the order of acquisition of locative relations in speech.

### 3.2 Spatial expressions informative in speech versus informative with gesture

**Fig. 5:**
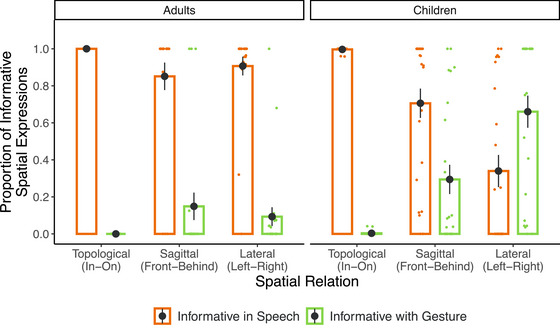
Proportion of spatial expressions informative in speech versus informative with gesture across spatial relations and age groups. *Note*. Bars and black dots represent group means. Error bars represent standard error of the group means. Colored dots represent participant means. Due to the binary nature of the data displayed in this figure, the proportion of expressions informative in speech and informative with gesture add up to 1.

Next, we focused on the informative descriptions only (91.7% of all descriptions; 95.4% for adults, 88.1% for children) and compared the descriptions that were already informative in speech to descriptions that become informative with gesture across spatial relations in adults and children (Fig. 5). We tested if children—but not adults—would be more likely to rely on gesture than speech to informatively express more complex spatial relations as opposed to less complex spatial relations.

When participants informatively expressed topological relations, adults exclusively (*M* = 1.00, *SE* = 0) and children almost exclusively (*M* = 0.996, *SE* = 0.002) used expressions that were already informative in speech. In other words, adults never described topological relations using expressions that became informative with gesture; children did so though extremely rarely (0.4% of the descriptions of topological relations). Because of this, the data from topological relations did not have sufficient variability and were excluded from the model. For completeness, these data are included in Fig. 5.

The remaining data were analyzed with a *glmer* model that tested the fixed effects of Spatial Relation (sagittal, lateral) and Age (adults, children) on the binary dependent variable at the trial level (1 = informative with gesture; 0 = informative in speech). Fixed effects of Age and Spatial Relation were tested with centered contrasts (−1/2, +1/2). The model included random intercepts for Items and random slopes for Spatial Relation by Subjects. The model revealed a significant interaction between Age and Spatial Relation (*β* = 7.388, SE = 3.106, *z* = 2.378, *p* = 017). As expected, children used descriptions that become informative with gesture more frequently for lateral relations than for sagittal relations (*β* = 8.399, SE = 2.085, *z* = 4.029, *p* < 001). Adults frequently used expressions that were already informative in speech and this did not change across Left‐Right and Front‐Behind (*β* = 1.011, SE = 2.135, *z* = 0.473, *p* = 636). Furthermore, for both types of spatial relations, children used descriptions that become informative with gesture more frequently than adults did (sagittal: *β* = 4.897, SE = 2.119, *z* = 2.311, *p* = 021; lateral: *β* = 12.285, SE = 2.869, *z* = 4.282, *p* < 001); though the developmental difference between children and adults was larger for lateral relations. Thus, as the complexity of the spatial relation increased, the main modality used for informatively expressing the spatial relation switched from speech to gesture for children but not for adults.

### 3.3 Informativeness (in speech and with gesture) of all spatial expressions

The final analyses focused on all spatial expressions. That is, in contrast to the previous model that only focused on the distribution within informative expressions, the final model also included descriptions that were not informative. Further, informative descriptions consisted of both descriptions that were already informative in speech (Fig. 4) plus descriptions that became informative with gesture. We compared the frequency of informative expressions across age groups and spatial relations. Of interest was whether the developmental differences between adults and children in the frequency of informative spatial expressions would diminish when gesture was considered together with speech and whether this decrease would be modulated by the complexity of the spatial relations. If so, developmental differences may disappear completely for less complex sagittal relations but may still persist for more complex lateral relations.

**Fig. 6:**
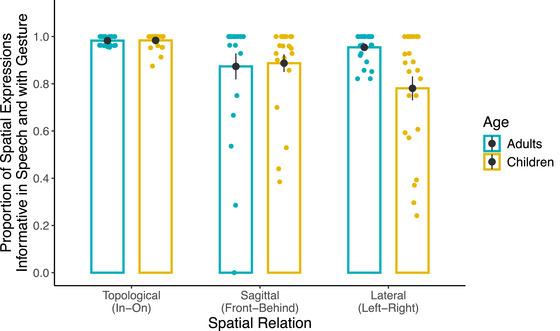
Proportion of all informative spatial expressions across spatial relations and age groups. *Note*. Bars and black dots represent group means. Error bars represent standard error of the group means. Colored dots represent participant means.

Fig. 6 shows the proportion of informative spatial expressions out of all spatial expressions for topological, sagittal, and lateral relations across adults and children. A *glmer* model tested the fixed effects of Spatial Relation (topological, sagittal, lateral) and (adults, children) on binary values for the presence of informative spatial expressions (1 = present, 0 = absent) at the trial level as the dependent variable. The same contrast coding strategies were used to test the fixed effects of Age and Spatial Relation in the analysis of the speech data described above. The model also included random intercepts for Subjects and Items. A more complex model that also included random slopes for Spatial Relation by Subjects produced a singular fit error. Thus, this term was omitted.

The model revealed that the interaction between Age and Spatial Relation was significant for both contrast levels (Topological vs. Sagittal/Lateral: *β* = −1.260, SE = 0.490, *z* = −2.569, *p* = 010; Sagittal vs. Lateral: *β* = −1.912, SE = 0.332, *z* = −5.761, *p* < 001). Follow‐up tests of pairwise comparisons with corrections for multiple comparisons revealed that when we considered both speech and gesture, adults and children produced informative spatial expressions equally frequently for topological (*β* = −0.318, SE = 0.669, *z* = −0.470 *p* = 640) and sagittal (*β* = 0.014, SE = 0.532, *z* = 0.030, *p* = 980) relations. Thus, developmental differences in the expression of sagittal relations in speech disappeared. For lateral relations, however, children produced informative spatial expressions still less frequently than adults (*β* = 1.898, SE = 0.526, *z* = 3.61 *p* < 001). Thus, even when gesture was considered together with speech, children continued to have difficulty mapping lateral relations onto spatial expressions.

## 4 Discussion

Previous research has shown that the emergence of locative terms in speech follows a lengthy yet cross‐linguistically stable timetable (Johnston, 1984; Johnston & Slobin, 1979). Traditionally, this timetable has been attributed to the complexity of the spatial relations expressed by these terms (Clark, 1973b, 1980). Our goal here was to explore an additional factor involved in this timetable. Specifically, we focused on the mapping difficulties introduced by the affordances of the modality used for expressing spatial information. We tested whether co‐speech gesture that exploits a different modality of expression than speech reduces the mapping difficulties and whether this was sensitive to the complexity of the spatial relations. Secondarily, we asked whether the developmental picture changes when spatial expressions in gesture are also considered when estimating the development of children's expressions of locative relations. Below, we summarize our main findings and discuss their implications for spatial language development, its relation to complexity of spatial relations, and the role of modality of expression in this relation.

As a first step, we assessed whether spatial descriptions in speech would follow previously established developmental patterns in the order of emergence of spatial terms. As expected, children's use of informative spatial expressions was modulated by the complexity of the spatial relations. Children described object locations informatively in speech most frequently—and in similar frequencies as adults—for the least complex topological relations. This was followed by sagittal relations, and then by lateral relations that were the most complex. Furthermore, when describing object locations that involved viewpoint‐dependent relations (front‐behind and left‐right), children produced informative expressions less frequently than adults. In both cases, when their speech did not informatively express the spatial relation, children tended to use locative terms corresponding to *side* or *next to*. These findings replicate the order of emergence patterns reported in previous developmental work (e.g., Abarbanell & Li, 2021; Durkin, 1980; Grigoroglou et al., 2019; Johnston, 1984; Levine & Carey, 1982), including work with Turkish‐learning children (Johnston & Slobin, 1979; Sümer, 2015; Sümer et al., 2014). Importantly, these developmental patterns were established by separate studies conducted on independent groups of children, using different stimuli and with slightly different methodologies. Our findings extend these previous reports by directly comparing three types of spatial relations varying in complexity within the same group of children and within a single paradigm using carefully matched stimuli.

Unlike children, adults overwhelmingly produced informative spatial expressions in their speech. Nevertheless, somewhat surprisingly, they produced informative expressions less frequently for both types of viewpoint‐dependent relations (front‐behind and left‐right) than topological relations. This was especially unexpected for sagittal relations. We speculate that this pattern might be attributed to the language‐specific strategies used for describing front‐behind relations in Turkish. Recall that in Turkish the locative terms corresponding to *side* are not reserved for left‐right relations but can also be used for describing front‐behind relations (Sümer et al., 2014). Further, in Turkish, there are a wider range of alternatives that correspond to *side* (e.g., *yan*, *taraf*) (Göksel & Kerslake, 2011). Thus, the difference between our findings and previous estimates from adults could be explained by the features of the specific languages from which these estimates are obtained. These features include, but may not be limited to, the number of terms available for expressing axial relations in a general way and how frequently used. Further work is needed to more precisely estimate the contributions of these language features in spatial language use in adults.

Even though children's speech patterns in our data follow an order consistent with the order of emergence reported in previous work, it might be somewhat surprising that the 8‐year‐old children in our sample did not use the terms corresponding to *front* and *behind* in similar frequencies as adults, given previous reports that these terms appear much earlier in speech (Johnston, 1984; Johnston & Slobin, 1979). One potential explanation for this pattern could be our coding system. Unlike prior work that has focused on the mere use of these terms in isolation, we focused on the use of these terms for accurate expression of the spatial relation between a figure and a ground object. In fact, one previous study also reports infrequent uses of *front* and *behind* in English‐speaking 3‐ to 5‐year‐olds based on similar criteria (Grigoroglou et al., 2019). Further, as mentioned above, another potential explanation has to do with the strategies available in Turkish for describing front‐behind relations. The fact that our adult participants sometimes used these alternative locative terms corroborates this explanation. This highlights the importance of drawing on evidence from typologically distinct languages when sketching the developmental timetable of the acquisition of spatial terminology.

Turning to multimodal expressions in speech and gesture, children frequently supplemented their underinformative spatial expressions in speech with spatial gestures. These findings are consistent with previous reports showing that children often rely on gesture to communicate information absent from the accompanying speech (Austin & Sweller, 2014; Calero et al., 2019; Göksun et al., 2010; Karadöller et al., 2024; Sauter et al., 2012; Sekine, 2009). However, such uses of gesture in our data were selective and depended on the complexity of the spatial relations. For the most complex left‐right relations, children were more likely to express the spatial information that disambiguated the relative locations of the objects in the gestural modality than expressing the spatial relation between the objects in speech. However, this pattern was reversed for sagittal relations that were relatively less complex than lateral relations. For the least complex topological relations, children were almost always already informative in speech. In other words, for children, gestures are used for different functions depending on the complexity of the spatial relations. Children used gestures in ways that increased the informativeness of spatial expressions only for the relations that involve a viewpoint and to a greater extent for lateral relations which are symmetrical and harder to distinguish from one another. Adults, on the other hand, were more likely to informatively express the spatial relation in speech regardless of the complexity of the relations. When they used gesture, they mostly used it for reinforcing the spatial meaning conveyed in speech and this function was stable regardless of the complexity of the spatial relation to be expressed. These findings suggest that the ability to combine the informativeness of different systems (speech vs. gesture) changes throughout development and across the complexity of the spatial relations.

Our findings also converge with recent developmental evidence on descriptions of object locations in sign. Recall that deaf signing children informatively express left‐right relations in sign more frequently than age‐matched peers do so in speech (Karadöller et al., 2024; Sümer, 2015). Nevertheless, even deaf signing children informatively describe left‐right relations less frequently than deaf signing adults. Thus, developmental differences between children and adults in expressing left‐right relations persist across speech, co‐speech gesture, and sign (Karadöller et al., 2024). Furthermore, even though visually motivated spatial expressions in sign are advantageous over locative terms in speech when expressing the most complex left‐right relations, there is no advantage of sign over speech when expressing less complex topological relations (in, on, under) (Sümer & Özyürek, 2020). Similarly, in our data when describing object locations involving in‐on relations, children produced informative expressions in speech in similar frequencies as adults. Thus, the developmental picture for topological relations did not change when we took into account spatial expressions in gesture. Together, these findings show that the facilitative role of visual modality in expressing spatial relations is sensitive to the complexity of the spatial relations.

Viewed within a broader discussion on how gesture interfaces with language and cognitive development, our findings cohere with evidence on children's multimodal explanations during problem‐solving across several domains (e.g., Piagegian conservation, math, Alibali et al., 2000; Alibali & Goldin‐Meadow, 1993; Broaders et al., 2007; Church & Goldin‐Meadow, 1986). This work revealed that although children seem to fail in problem‐solving tasks based on the information they convey in speech, their gestures express the correct solutions to the problems. Nevertheless, for gestures to reflect the conceptual knowledge not yet expressed in speech, children need to be in a state of transitional knowledge such that they have a partial understanding of a concept and are ready to learn and benefit from gestures (Goldin‐Meadow, Alibali, & Church, 1993; Goldin‐Meadow & Alibali, 2013). Similarly, to be able to rely on gestures when describing object locations, children need to have a certain understanding of the spatial relation between the objects at the cognitive level. When their cognitive understanding of the spatial relation is not yet at that level—as in the case of left and right—the facilitative role of gesture is also limited. However, they can fully benefit from gestures when their cognitive understanding of spatial relations is more complete—as in the case of front and behind.

The present study has some limitations that open up directions for future research. A first issue concerns the developmental differences between children and adults. Children (and adults) were at ceiling and almost always informatively expressed topological relations in speech. Further, when both speech and gesture were considered, children overwhelmingly produced informative expressions when describing sagittal relations. This was expected based on the age range of our child participants and on previous work. However, it is also possible that in younger age groups the developmental differences between children and adults still persist for sagittal relations even with gestures. Future work with children younger than 8 can address this possibility. Second, the present study used a confederate addressee who was always an adult. The inclusion of an addressee who did not see the target picture and had a clear communicative goal was a methodological improvement. This helped us establish how children and adults describe spatial relations under controlled circumnutates that still approximate naturalistic communication by having genuine informational needs. It is also plausible that children's and adults’ spatial expressions change based on addressee characteristics. Future versions of this work can address this issue by including a naïve familiar interlocutor or by matching the child‐adult status of the speaker and the addressee. Finally, the majority of the empirical work, on how gesture relates to language and cognitive development, focuses on group‐level differences. However, a growing body of work suggests that there are individual differences in gesture use (Özer & Göksun, 2020). Future work can include measures of cognitive and linguistic skills or other background factors to extend this line of work to spatial language. A particularly relevant variable for spatial gestures is literacy levels, as it predicts the dominant axes used for metaphorical spatial gestures about time (Casasanto & Jasmin, 2012; Stites & Özçalışkan, 2021). It remains to be seen if individual differences in literacy levels are also related to changes in the expression of literal spatial meanings in gesture.

## 5 Conclusions

The present study offers novel evidence that the acquisition of spatial expressions is jointly shaped by the complexity of the spatial relations and the modality of expressions. Children selectively rely on speech versus co‐speech gesture to express spatial relations when describing object locations. Further, gesture eases the expression of spatial meaning not yet expressed in speech; but this facilitative role of gesture is dependent on the complexity of the spatial relations. Finally, the present study offers new possibilities for future work by showing that a multimodal approach that considers both spoken and gestural expressions of spatial relations more realistically approximates children's language and cognitive development in this domain.

## Funding

This research was supported by NWO‐VICI grant 277‐70‐013 awarded by The Dutch Research Council to A.Ö.

## Competing interests

The authors declare no competing interests.

## Supporting information



Supporting Information

## Data Availability

The data that support the findings of this study are available in Open Science Framework at https://osf.io/njufw/.

## Notes

1 Sample size was determined based on the following constraints. Data reported in this article were collected as part of a larger project that also involves comparisons between deaf children acquiring Turkish Sign Language and hearing children acquiring spoken Turkish, as well as adult controls from each population. For deaf participants, we collected data from all children attending the deaf schools in Istanbul, Turkey whose parents provided consent and met our selection criteria (i.e., having been exposed to a sign language from birth by their deaf parents, not having any developmental disorders). The number of hearing children and adults was matched to the number of deaf signing children and adults.

2 A more complex model that also included type of display (contrastive vs. noncontrastive) as a fixed effect did not fit the data better in this and the subsequent analyses. Therefore, the data from the two types of displays were collapsed for the analyses. Results and analysis code can be found at: https://osf.io/njufw/.
