# Supplementary material for: Gesture Reduces Mapping Difficulties in the Development of Spatial Language Depending on the Complexity of Spatial Relations
Source: Cogn Sci. 2025 Feb 24;49(2):e70046. doi: 10.1111/cogs.70046 (PMC11849910; doi:10.1111/cogs.70046)
Supplement: Supplementary file 1 — Supporting Information [file COGS-49-e70046-s001.docx]

**List of Stimuli for the Description Task**

|  | **Target Picture (Ground, Figure, Relation)** | **Target Relation** | **Non-target Relations** |
| --- | --- | --- | --- |
| **1** | plate cheese in | Topological (in) | left, right, front |
| **2** | food box plastic paintbrush in | Topological (in) | left, behind, front |
| **3** | box leaves in | Topological (in) | front, behind, on |
| **4** | bag postcard in | Topological (in) | front, behind, on |
| **5** | envelope pills in | Topological (in) | front, behind, on |
| **6** | hat green ruler in | Topological (in) | left, behind, on |
| **7** | pan green pepper in | Topological (in) | left, behind, front |
| **8** | shoe lighter in | Topological (in) | left, behind, on |
| **9** | box magnifier in | Topological (in) | right, behind, front |
| **10** | food box black notebook in | Topological (in) | left, right, behind |
| **11** | envelope key in | Topological (in) | right, behind, on |
| **12** | mug wooden paintbrush in | Topological (in) | front, behind, on |
| **13** | cooking pot red pepper in | Topological (in) | left, right, front |
| **14** | plate red brush in | Topological (in) | right, behind, front |
| **15** | tray ball on | Topological (on) | left, right, front |
| **16** | shoe camera on | Topological (on) | front, behind, in |
| **17** | shoe sock on | Topological (on) | front, right, in |
| **18** | box red pencil case on | Topological (on) | left, right, behind |
| **19** | slipper glasses on | Topological (on) | front, right, in |
| **20** | paper coke can on | Topological (on) | right, front, behind |
| **21** | book garlic on | Topological (on) | left, right, behind |
| **22** | envelope bracelet on | Topological (on) | front, behind, in |
| **23** | tray white candle on | Topological (on) | right, front, behind |
| **24** | book duster on | Topological (on) | left, front, behind |
| **25** | hat water bottle on | Topological (on) | front, behind, in |
| **26** | cooking pot pink spatula on | Topological (on) | left, front, in |
| **27** | paper rubber on | Topological (on) | left, right, front |
| **28** | pan plastic spoon on | Topological (on) | left, front, in |
| **29** | cooking pot blue brush front | Sagittal (front) | right, on, in |
| **30** | tray small bottle front | Sagittal (front) | left, right, on |
| **31** | food box pink flower front | Sagittal (front) | behind, right, in |
| **32** | pan eggplant front | Sagittal (front) | behind, left, in |
| **33** | plate red napkin front | Sagittal (front) | left, right, in |
| **34** | plate paring knife front | Sagittal (front) | behind, left, in |
| **35** | bag pink perfume bottle front | Sagittal (front) | left, in, on |
| **36** | envelope nail clipper front | Sagittal (front) | left, in, on |
| **37** | shoe red board marker front | Sagittal (front) | behind, on, in |
| **38** | slipper black perfume bottle front | Sagittal (front) | behind, on, in |
| **39** | book pacifier front | Sagittal (front) | left, right, on |
| **40** | hat orange notebook front | Sagittal (front) | behind, on, in |
| **41** | box hairdryer front | Sagittal (front) | left, right, in |
| **42** | mug spoon front | Sagittal (front) | right, on, in |
| **43** | mug credit card behind | Sagittal (behind) | left, on, in |
| **44** | cooking pot metal ladle behind | Sagittal (behind) | front, on, in |
| **45** | paper pen behind | Sagittal (behind) | front, left, on |
| **46** | tray apple behind | Sagittal (behind) | front, left, on |
| **47** | book board marker behind | Sagittal (behind) | front, right, on |
| **48** | food box black pencil case behind | Sagittal (behind) | left, right, on |
| **49** | paper onion behind | Sagittal (behind) | front, right, on |
| **50** | mug yellow scissors behind | Sagittal (behind) | left, right, on |
| **51** | pan wooden spatula behind | Sagittal (behind) | front, on, in |
| **52** | hat green umbrella behind | Sagittal (behind) | right, on, in |
| **53** | bag red giftbox behind | Sagittal (behind) | right, on, in |
| **54** | slipper metal giftbox behind | Sagittal (behind) | left, right, in |
| **55** | slipper cell phone behind | Sagittal (behind) | front, on, in |
| **56** | bag headphones behind | Sagittal (behind) | front, on, in |
| **57** | cup chocolate left | Lateral (left) | right, behind, on |
| **58** | jar watch left | Lateral (left) | behind, on, in |
| **59** | plant pot sponge left | Lateral (left) | right, behind, in |
| **60** | basket flute left | Lateral (left) | behind, on, in |
| **61** | vase banana left | Lateral (left) | right, front, on |
| **62** | vase metal scissors left | Lateral (left) | behind, in, on |
| **63** | bowl tree branch left | Lateral (left) | front, in, on |
| **64** | moneybox orange balloon left | Lateral (left) | right, front, on |
| **65** | plant pot torch left | Lateral (left) | front, on, in |
| **66** | bowl wooden ruler left | Lateral (left) | right, behind, on |
| **67** | basket toilet paper left | Lateral (left) | right, front, in |
| **68** | jar mandarin left | Lateral (left) | right, front, in |
| **69** | moneybox straw left | Lateral (left) | front, on, in |
| **70** | cup pencil left | Lateral (left) | front, in, on |
| **71** | vase red flower right | Lateral (right) | front, on, in |
| **72** | basket butter knife right | Lateral (right) | front, in, on |
| **73** | bowl metal spoon right | Lateral (right) | behind, on, in |
| **74** | moneybox money right | Lateral (right) | front, on, in |
| **75** | vase blue candle right | Lateral (right) | left, behind, in |
| **76** | cup egg right | Lateral (right) | left, front, in |
| **77** | basket remote right | Lateral (right) | left, behind, on |
| **78** | jar lemon right | Lateral (right) | front, in, on |
| **79** | plant pot iPhone right | Lateral (right) | left, front, on |
| **80** | moneybox stone right | Lateral (right) | left, front, on |
| **81** | jar screwdriver right | Lateral (right) | left, behind, on |
| **82** | plant pot fan right | Lateral (right) | behind, in, on |
| **83** | cup fork right | Lateral (right) | behind, on, in |
| **84** | bowl tomato right | Lateral (right) | left, front, in |

**Instructions for the Description Task**

**Instructions in Turkish**

*Bu oyunda ektranda dört tane resim göreceksin. Her resimde iki tane nesne var.*

*Sonra bu resimlerden birine işaret eden bir ok çıkacak. Daha sonra ok ve resimler kaybolacak, gri bir ekran gelecek.*

*Senden istediğim gri ekran geldiğinde okun işaret ettiği resmi [dinleyicinin adı]’ya anlatman. Onun ekranında ok yok, sadece resimler var. O da senin anlattığına göre doğru fotoğrafı seçecek.*

*Anlatman bitince ENTER’a basıp sonraki resimlere geçebilirsin.*

*Şimdi birkaç örnek yaparak başlayalım!*

**English translation**

*In this game you will see four pictures on your screen. There are two objects in each picture.*

*Then an arrow will appear pointing to one of these pictures. Then the arrow and the pictures will disappear and a gray screen will appear.*

*Your task is to describe the picture that the arrow points to [name of the addressee] when the gray screen appears. There is no arrow on their screen, only pictures. They will choose the right picture according to your description.*

*When you are done describing you can press ENTER and move on to next pictures.*

*Let's start with a few examples!*
